# Identification and antimicrobial susceptibility of acute external otitis microorganisms

**DOI:** 10.1016/S1808-8694(15)30598-X

**Published:** 2015-10-18

**Authors:** Janaina Cândida Rodrigues Nogueira, Margareth de Fátima F. Melo Diniz, Edeltrudes Oliveira Lima, Zilka Nandes Lima

**Affiliations:** 1 MsC. ENT Physician.; 2 PhD; Director - Centro de Ciências da Saúde/ UFPB.; 3 PhD, Professor of Mycology - UFPB.; 4 Pharmacist/ Biochemist - Laboratório Hemato.

**Keywords:** culture media, microbiology, otitis

## Abstract

Acute external otitis is a polymicrobial infectious disease.

**Aim:**

The purpose of this study was to isolate, identify and determine the Antimicrobial susceptibility of organisms causing otitis externa (OE).

**Methods:**

twenty-seven swabs were taken from the ears of 27 patients with OE for culture and 22 microorganisms were isolate to evaluate sensitivity. In vitro susceptibility tests were performed by agar diffusion disk and results were interpreted according to Clinical Laboratory Standards.

**Results:**

10 Ear cultures were positive for *S. aureus*, 8 for *P. aeruginosa*, 5 for *P. aeruginosa* and *S. aureus* and 4 for fungal organisms (*Candida albicans* e *C. Krusei*). Gentamicin and quinolones were active against all bacteria tested and significant resistance to amoxicillin/clavulanate was observed. The tested species of *Candida* had been sensitive to amphothericin B, nystatin, fluconazole and clotrimazole and resistant to miconazole.

**Conclusion:**

Acute external otitis is a polymicrobial infection and proper knowledge regarding microorganism etiology and susceptibility will contribute to rational antibiotic usage and treatment success.

## INTRODUCTION

Otitis externa is an inflammation on the skin of the external auditory canal, usually associated with secondary bacterial and/or fungal infection of macerated skin and subcutaneous cellular tissue. Many factors act, changing the most superficial skin layers, opening doors for infection to happen, making bacterial otitis externa the major cause of disease in the external ear^1^. Systemic conditions such as anemia, low vitamin concentration in the body, endocrine disorders - especially diabetes and many forms of dermatitis such as seborrhea, psoriasis and eczema may reduce resistance to infections in the external auditory canal, causing the development of otitis externa^2^.

To treat this disease we use ear drops with antibiotics and/or antifungal agents associated with anesthetic and/or anti-inflammatory agents, and systemic drugs are indicated should complications occur^3^. The goal of this study was to isolate and identify species of microbes in patients with clinical diagnosis of acute otitis externa, and the susceptibility of microorganisms to the antimicrobial drugs standardized in the antibiogram test.

## MATERIALS AND METHODS

The patients were seen in public and private otorhinolaryngology medical offices. Twenty seven patients diagnosed with acute otitis externa were selected regardless of age and gender. We used Goldenber's inclusion criteria^4^, which are: a diagnosis of otitis externa, no prior treatment and intact ear drum. The patients selected had material collected from the diseased ear by means of a swab by a trained professional and the material collected was transported in Stuart's culture medium. The study was carried out following the regulatory standards and guidelines for research involving human beings, Resolution # 196/1996^5^ from the Ministry of Health and approved by the Ethics in Research Committee of the Lauro Wanderley University Hospital, protocol # 341, and the patient signed an informed consent form in order to be submitted to material collection and culture.

The cultures were randomly sent to three different labs and the in vitro susceptibility tests were carried out through the disc spread agar, and the results were interpreted following conventional clinical and laboratorial standards^6^, ^7^, ^8^.

## RESULTS

**Figure 1:**
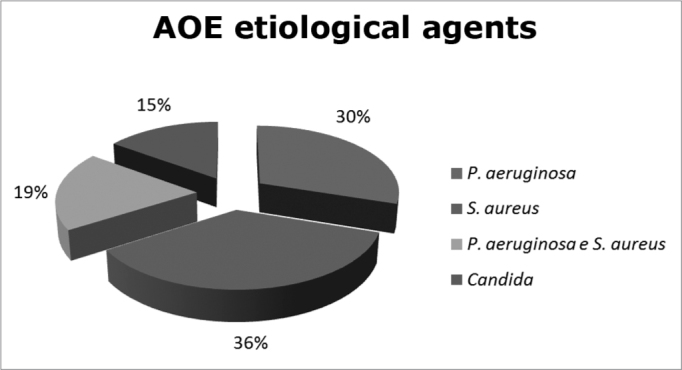
Distribution of acute otitis externa etiological agents.

The patients were seen in an otorhinolaryngology ward, regardless of age and gender, and were selected through clinical diagnosis, twenty seven patients with acute otitis externa. They had material collected from their diseased ear, and this material was submitted to bacterial culture and antibiogram study. *Staphylococcus aureus* were seen in 10 cultures (37%), *Pseudomonas aeruginosa* in 8 cultures (29.6%), *Pseudomonas aeruginosa* and *Staphylococcus aureus* together were seen in 5 cultures (18.5%), and *Candida* fungus was found in 4 cultures, (14.9%), in all cases, they were associated to Gram positive and Gram negative bacteria. This data is shown on Figure 1.

From the twenty seven cultures obtained in the present study, we isolated eight *S. aureus* strains, twelve *P. aeruginosa* strains, one *Candida albicans* strain and one *C. krusei* strain. The results from the susceptibility test for *S. aureus* can be seen on Table 1, for *P. aeruginosa*, on Table 2; and for fungii on Table 3.

**Table 1:** Assessment of *S. aureus* strains obtained from patients with AOM in terms of their susceptibility to antimicrobial agents. S= sensitive - R= resistant i= intermediate x= not tested

	Strain 1	Strain 2	Strain 3	Strain 4	Strain 5	Strain 6	Strain 7	Strain 8	Sensitivity %
Quinolones	S	S	S	S	S	S	S	S	100
Macrolides	S	R	R	R	R	R	R	S	25
Penicillin	S	R	R	R	R	R	R	R	12,5
Augmented Amoxicillin	S	S	S	R	S	S	R	S	75
Ampicillin	S	R	R	R	R	R	R	R	12,5
Amoxicillin	S	R	R	R	R	R	R	R	12,5
Cephalosporin1	S	S	S	S	S	S	S	X	100
Cephalosporin2	S	S	S	S	S	S	S	S	100
Cephalosporin 3	S	S	S	S	S	S	S	X	100
Aminoglycoside	S	S	S	S	S	S	S	S	100
Chloramphenicol	S	S	S	S	S	S	S	S	100
Tetracycline	S	S	R	R	S	S	X	X	50
Vancomycin	S	S	S	S	S	S	S	S	100
Clindamycin	S	R	S	R	R	R	R	X	25

**Table 2:** Antibiogram from *Pseudomonas* aeruginosa strains taken from patients with AOM. S= sensitive - R= resistant i= intermediate x= not tested

	Strain 1	Strain 2	Strain 3	Strain 4		Strain 6	Strain 7	Strain 8	Strain 9	Strain 10	Strain 11	Strain 12	Sensibi lidade %
Quinolones	S	S	S	S	S	S	S	S	S	S	S	S	100
Erythromycin	R	R	R	R	R	R	R	R	R	R	R	R	-
Augmented Amox.	R	R	R	R	R	R	R	R	R	R	R	R	-
Ampicillin	R	R	R	R	R	R	R	R	R	R	R	R	-
Amoxicillin	R	R	R	R	R	R	R	R	R	R	R	R	-
Ceftazidime	I	I	S	I	S	I	S	I	I	I	I	I	25
Cefotaxime	I	I	S	I	S	I	S	I	I	I	I	I	25
Ceftriaxone	I	I	S	I	S	R	S	I	I	I	I	I	25
Lincomycin	R	R	R	R	R	R	R	X	X	R	R	R	-
Tobramycin	S	S	S	S	S	X	S	S	S	S	X	X	100
Chloramphenicol	R	R	I	R	I	R	R	R	R	R	R	R	-
Tetracycline	R	R	R	R	R	R	R	R	R	R	R	R	-
Piperacillin/Azobatan,	S	S	S	S	S	R	S	S	S	S	S	S	91
Aztreonam	S	S	S	S	S	R	S	S	S	S	S	S	91
Imipenen, Meropenem	S	S	S	S	S	S	S	S	S	S	S	S	100

**Table 3:** Antibiogram of the *Candida* strains in AOE patients. S= sensitive - R= resistant i= intermediate x= not tested

	*Candida albicans*	*Candida krusei*
Miconazole	R	R
Ketoconazole	I	I
Fluoconazole	I	S
Clotrimazole	S	I
Anphotericin B	S	I

## DISCUSSION

Otitis externa is one of the most commonly found diseases in otolaryngology wards, especially during the Summer time; affecting about 10% of the population^9^, regardless of gender and age^10^. It may be localized or diffused, acute or chronic^11^. The most commonly found bacterial agents are *P. aeruginosa* and *S. aureus*; and as far as fungii are concerned, the agents are *Aspergillus* and *C.albican*s^2^, ^11^.

The results hereby achieved lead us to conclude that the microorganisms isolated and identified on the external auditory canal skin were *P. aeruginosa* and *S. aureus* bacteria and *Candida* fungii, and considering the latter, *Candida kruse*i was also found, which is not very frequently reported as a microbial agent associated with otitis; and this data is similar to what is found in the literature^12^.

Hwang et al.^13^ carried out a bacteriology study in 161 patients and they observed that *Staphylococcus aureu*s was as frequent as *Pseudomonas aeruginosa* in otitis externa patients. This data rectify the findings of the present investigation, because in our study there was no statistically significant difference between *P. aeruginosa* and *S. aureus.*

Although *P. aeruginosa* is the most frequently found bacteria in otitis externa, the percentage of positive cultures for *S. aureus* was very significant in numerous papers published, establishing both agents as the main ones responsible for infections in the external ear. In the present investigation there was a subtle predominance of *S. aureus* in relation to *P. aeruginosa*, and they were found together in 18.5% of the cases, which is very similar to what is found in the literature in regards of polymicrobial flora^12^.

The treatment of acute otitis externa is based on local debridement, use of ear drops with acidifying agents and/or antimicrobial agents and, in cases of intense edemas and/or purulent secretions, one would use oral anti-inflammatory and antimicrobial agents. The use of parenteral antimicrobial agents is indicated in cases of malignant otitis externa.

The most commonly used ear drops have in their formulas: antibiotics, hormonal anti-inflammatory agents and, in some products, anesthetics. The most used antibiotics are: aminoglycosides (gentamicin and neomycin), polymyxin B, chloramphenicol and quinolones (ofloxacin, ciprofloxacin).

Since the introduction of fluoroquinolones in the medical practice, in 1980, they have been successfully used in numerous infections, especially those caused by *P. aeruginosa*, being added to the weaponry of ear drops^14^.

Ofloxacin and ciprofloxacin which are available as ear drops and, ciprofloxacin, also in ophthalmic solutions, can also be used to treat the auditory canal. The greatest advantage of these agents is the absence of ototoxic effects. Despite the systemic use of fluoroquinolones be usually contraindicated in children, topical solutions are safe and efficatious^14^.

Gentamicin and polymyxin B were the most effective ear drops to treat acute otitis externa against *P. aeruginosa*, according to a study carried out by Loh et al.^15^, in Singapore, with 107 patients. In the present investigation, all the *P. aeruginosa* strains exposed to gentamicin proved to be sensitive. However, we did not test polymyxin B, since the labs were not standardized to test this drug in the antibiogram.

In assessing the susceptibility of *P. aeruginosa* strains taken from patients with acute otitis externa, three were sensitive and nine had intermediate sensitivity to ceftazidime, while all the 12 strains were sensitive to ciprofloxacin. Two strains were resistant and one strain had intermediate sensitivity to neomycin, we did not observe resistance to fluoroquinolones; and all the strains exposed to gentamicin were sensitive.

Chloramphenicol is also used as ear drops; however, in the present investigation the high resistance rate of *P. aeruginosa* towards this drug in the susceptibility test, including augmented amoxicillin, these drugs used per os for ear diseases, as first line options, especially in the treatment of otitis media, must not be considered in the oral treatment of otitis externa, according to this susceptibility study, which share the same resistance findings from other papers^11^, ^16^.

As far as *S. aureus* is concerned, chloramphenicol was very active, as were quinolones, fluoroquinolones, neomycin and oxacillin, and one strain was resistant to gentamicin.

There was resistance to amoxicillin; however, when associated with augmented amoxicillin, its action was satisfactory. Second and third generation cephalosporins were active against the bacteria tested, and it can be used as a treatment option. It is important to notice that because of the diversified germ flora and variable susceptibility, in the complication of otitis externa it is paramount to order an antibiogram test in order to achieve a more efficient treatment.

There have been numerous progresses in the treatment of fungal infections with systemic antifungal drugs, such as polyenes (oral nystatin and intravenous amphothericin B) and imidazole agents (miconazole - oral and IV and oral ketoconazole). Ear drops with antifungal agents, especially those with imidazole agents have excellent action in otolaryngology; however, since otitis media is usually caused by a polymicrobial flora, with fungi and bacteria together, it would be very important to use topical agents with broad spectrum^17^.

There are very few ear drops to treat otomycosis today in the market, usually we have to use dermatologic creams or lotions for its treatment. The present study isolated two strains of *Candida*, a fungus very much found in otomycosis, in all cases associated to gram positive and gram negative bacteria. Since it is usual to find a polymicrobial flora in otomycosis, it would be better to use a topical medication with antifungal and antibacterial agents, which is not very much found in clinical practice.

The *Candida* species found in this study were all sensitive to Amphothericin B, nystatin, fluconazole and clotrimazole and resistant to miconazole.

Otomycosis represents a small percentage of the clinical otitis externa and, in contrast with external bacterial otitis, there are not too many specific topic preparations for its treatment.

We carried out an in vitro study of the susceptibility of the fungii responsible for otomycosis and we observed a good antifungal activity of clotrimazole and nystatin. As far as nystatin is concerned, such data match the ones found in the present investigation, since the *Candida* species were all susceptible to these antifungal medication^18^.

Imidazole derivatives, miconazole and clotrimazole may be successfully used in otomycosis in topical solutions^17^. However, in the present investigation the two strains were resistant to miconazole and one strain had intermediate sensitivity to clotrimazole. Among imidazole derivatives, ketoconazole represents a revolutionary antifungal agent, especially against *Candida albicans*, besides having low toxicity^17^; however, in the present study it showed intermediate sensitivity.

One important finding from the present investigation was that some antibiotic agents which are part of ear drops were not tested in some antibiograms, in other words, they were not part of the laboratory routine and there were no standardizing for the antibiotic agents that were tested.

## CONCLUSION

Acute otitis externa had a polymicrobial etiology, with a predominance of *S. aureus*, *P.aeruginosa* and *Candida*, The bacteria assessed were resistant to penicillin and augmented amoxicillin and had good sensitivity to most antifungal agents. Therefore, a better knowledge of the etiology and the susceptibility of the microorganisms responsible for the acute otitis externa contributes to the judicial use of antimicrobial agents and treatment success.
